# Higher education personnel’s perceptions about telepresence robots

**DOI:** 10.3389/frobt.2022.976836

**Published:** 2022-12-06

**Authors:** Janika Leoste, Sirje Virkus, Aleksei Talisainen, Kalle Tammemäe, Katrin Kangur, Izabella Petriashvili

**Affiliations:** ^1^ Tallinn University of Technology, Tallinn, Estonia; ^2^ School of Digital Technologies, Tallinn University, Tallinn, Estonia; ^3^ Department of Education Sciences, Faculty of Psychology and Education Sciences, Tbilisi State University, Tbilisi, Georgia

**Keywords:** distance learning, telepresence robots, perceptions, higher education, literature review

## Abstract

The interest towards using telepresence robots in a variety of educational contexts is growing, as they have a great potential to enhance the educational experience of remote learners and provide support for teachers. This paper describes a study, examining the perception of Georgian university personnel about the use of telepresence robots in education. This exploratory research aimed to obtain evidence-based information on how the personnel (16 persons) from eight Georgian universities perceived the telepresence robots’ role in enhancing learning and teaching, and what challenges, benefits, opportunities, weaknesses and threats would characterise these robots. The results of the study revealed that the university personnel perceived telepresence robots to have a great potential to enhance educational activities. In addition, the participants indicated the major challenges, benefits, opportunities, weaknesses and threats, regarding integrating telepresence robotics into the teaching and learning in Georgia. Recommendations for future research are also presented.

## 1 Introduction

The global COVID-19 pandemic has increased the demand for remote working technologies in the education sector, especially due to the emergence of new viable virtual and hybrid learning models that combine in-person classroom learning with remote learning from home (Davey, 2021; Keller et al., 2021; Khadri, 2021). Since reduction of personal contact is the key in fighting the COVID-19 pandemic, the importance of remote communication solutions is growing. Next to the more common forms like video and audio conference calls, Telepresence Robots (TPRs) are becoming more popular (Perez, 2020; Davey, 2021; Keller et al., 2021). Yet, implementing emerging technologies effectively is a difficult task when people lack related knowledge, skills and readiness to apply them, or have sceptical and negative attitudes towards technology (Leoste et al., 2021a). Convincing teachers to accept and adopt novel technologies requires good understanding about introduction of technologies to teachers, teachers’ technological frames, gathering their feedback, and constructing an implementation plan that considers their needs (Leoste et al., 2021b; Spieth et al., 2021).

This study examines the perspectives of implementing TPRs in higher education institutions. The main goal of the study is to understand the attitudes of personnel from eight Georgian universities towards TPRs *via* analysing their first-hand perceptions from a workshop conducted in May 2022. The research process was guided by the following Research Questions (RQs):

RQ1: What is the current state of using TPRs in education?

RQ2: What are university personnel’s perceptions about TPRs?

RQ3: What are the benefits, opportunities, weaknesses, threats and challenges of TPR proliferation, from the point of view of university personnel?

TPRs are defined in this research as mobile robots, which are designed for modern remote communication. They are equipped with cameras, microphones, a screen, and sensor-assisted motion control. TPRs can enable their operators to feel more present at the remote location, as they can move freely without being dependent on another person when turning camera view (Keller et al., 2021).

section 2 of this paper covers the literature that provides the theoretical background for this study, also answering RQ1. In section 3, the methods for research, data collection and analysis are discussed. The findings of the study are presented in section 4. In section 5 the findings are presented in connection with the previous research, together with conclusions, and discussing the limitations of the study and areas for future research.

## 2 Literature review

A search in the Web of Science database in June 2022, using the keyword ‘*telepresence robots*’ in the topic area, provided 190 results from the fields of computer science (50%), robotics (39%), engineering (28,4%), automation control systems (6.8%) and education and educational research (6.3%). The first publication about TPRs in this database dates from 2006 and the number of publications has grown steadily. The main contributions come from the United States (37.4%), Japan (12.1%), Canada (7.9%), Australia (6.8%) and China (5.8%).

TPRs are used in or have potential applications in nearly all professional or commercial settings (Reis et al., 2018). The mobile robotic telepresence phenomenon was first studied by Paulos and Canny’s (1998) who researched the PRoP (*Personal ROving Presence*) system. Since then, many researchers have explored the use of TPRs in a variety of fields and contexts, such as medicine (Daruwalla et al., 2010), interpersonal communication (Ogawa et al., 2011), museums (Roberts & Arnold, 2012) and education (Kwon et al., 2010; Tsui et al., 2011; Tanaka et al., 2014; Conti et al., 2017).

In this literature review section, we reviewed the literature regarding the usage of TPRs in education. The literature review is structured around four main themes: 1) the nature of TPRs; 2) the use of TPRs in education; 3) the benefits of TPRs in education; and 4) the limitations, threats and challenges related to TPRs in education.

### 2.1 The nature of TPRs

The term “telepresence” was coined by Marvin Minsky, co-founder of the Artificial Intelligence Laboratory at the Massachusetts Institute of Technology (MIT) and one of the pioneers in this area of research, in 1980 in reference to teleoperation systems for manipulating remote physical objects (Rae et al., 2015). Telepresence is defined as the feeling of being fully present at a remote location from one’s own physical location. Telepresence creates a virtual or simulated environment of the real experience for the robot operator (El-Gayar et al., 2005). A TPR is a remote-controlled movable wheeled device, which is equipped with cameras, speakers, microphones, screen, and sensor-assisted motion control, and other interactive features, which are especially designed for communicating and collaborating remotely. A remote user can log into the robot to control it *via* smartphones, tablets, or computers, while experiencing the onsite surroundings on a screen. Simultaneously, the users’ face is being projected live onto the robots screen, allowing onsite personnel to communicate with the user in a face-to-face manner, establishing a remote telepresence (Perez, 2020).

Commercially available TPRs can perform a range of functions. Depending on the model, some TPR models allow changing the robot’s height by the remote user. This allows the user to set the robot’s size to match their own height. The head can move around from side to side and may have advanced features, for example, real-time full-resolution zoom, which provides UHD 4 K resolution of objects such as whiteboards. While some models have laser pointers, auto-navigation, and mapping features (Davey, 2021), options to manipulate objects in remote environments and get tactile feedback remain limited. Prices for TPR models vary from $1,500 (in education) to $1.5 million (in tele-surgery) each.

Thus, although the functions of TPRs vary depending on their type, they generally include the following features: 1) two-way audio communication with others in remote locations; 2) one-way or two-way video screens; 3) movement of the robot by the operator; 4) mobility control of the hands and head-equivalent parts; and 5) wireless internet connection (Takeuchi et al., 2020; Kaelin et al., 2021).

### 2.2 The use of TPRs in education

TPRs are becoming increasingly popular, having a great potential for sound pedagogical reasons within education at all levels (Conti et al., 2017; Reis et al., 2018). The popularity of TPRs is increasing due to their cost effectiveness, savings on energy and time, and enhanced communication and presence (Davey, 2021). In principle, telepresence should allow students and teachers to *attend classes remotely*, using a robotic body to represent them in the physical classroom. TPRs makes it possible for students to become involved in group-work and interact with their peers and lecturers under certain scenarios that could out rule their direct physical participation. These scenarios include chronic illnesses, medical conditions, hospitalisations, or disabilities that may require individual modifications to traverse around university (Weiss et al., 2001; Herring, 2013; Tanaka et al., 2013; Newhart et al., 2016; Weibel et al., 2020; Page et al., 2021; Lei et al., 2022).

Communicating *via* a TPR may enhance feelings of *social presence* in both the controller and the interaction partner (Lee & Takayama, 2011; Cha et al., 2017). Social presence was first conceptualised by Short et al. (1976) and was defined as the salience of the interactants and their interpersonal relationship during a mediated conversation. Later on, Garrison (2007, p. 2) describes social presence “as the ability to project one’s self and establish personal and purposeful relationships.” Research suggests that TPRs may enhance social presence between students and that the robot’s anthropomorphic characteristics may stimulate affection (Adalgeirsson & Breazeal, 2010).

TPRs can *compensate the lack of mobility* of students (i.e., distant residency, bad weather conditions, disabilities or illness, force majeure conditions such as epidemics) and enables them to study in a social environment, where they can actively participate in the class on the peer-to-peer basis (Wernbacher et al., 2022). By removing the need to travel physically to university, TPRs are making it easier for students and teachers to attend classes, even if they are unable to leave their homes (Khojasteh et al., 2019). In addition, TPRs can be used to provide education to *students in rural areas* who may not otherwise have access to quality education (Wernbacher et al., 2022).

TPRs have been used for students with *special needs*, such as those having autism, attention deficit hyperactivity disorder (ADHD) and diabetes (Yousif, 2021). Currently there are plenty of examples of robots already developed and commercialised with the goal of helping the students with special needs (Reis et al., 2018). Conti et al. (2017) affirms that the research in robotics has made numerous possibilities available for further innovation in the education of children, especially in the rehabilitation of those with learning difficulties and/or intellectual disabilities. They provide opportunities for making practical elements of the curriculum accessible for a wide range of disabled students (Reis et al., 2018). Thus, TPRs can be effectively deployed for students that are medically not able to visit a classroom (Weibel et al., 2020).

TPRs can also be used to *provide additional support* to students who have difficulty with a particular subject. For example, a TPR can be used outside of regular hours to provide additional individual instruction. These robots offer students a more personal and immersive experience than traditional online lessons. Bell (2017) argued that TPRs are game-changers due to their potential to enhance and improve educational experiences for remote learners and they have made the teaching and learning strategies much easier (Kennedy, 2016; Liao & Lu, 2018).

The TPRs can support *internationalisation* by enabling educational institutions to access experts from around the world to interact and teach students, enriching their educational and cultural experience. TPRs offer more interpersonal face-to-face interaction than regular MOOCs or videoconferences, and can potentially gain mass preference over regular online courses, and could become the standard for online learning (Joshi, 2021).

During the last decade, TPRs have gained more attention in universities, due to the need of teaching personnel to find new technologies and methods that enable them to better connect with distance students (Kennedy, 2016; Zhang et al., 2018; Corsby & Bryant, 2020; Khadri, 2021). For example, the Michigan State University has been experimenting with various types of TPRs in a hybrid program where students can attend the same classes in person and remotely since 2015. They used the Kubi and Double TPRs, in conjunction with video conferencing software. Both students and teachers were very positive about the use of TPRs and it was found that the technology has demonstrated its power to enhance remote learners’ educational experience in very important ways (Meyer, 2015; Bell, 2017; Cheung et al., 2018). The Duke University School of Nursing combined telepresence and simulation technologies. This allowed distance-based nurse practitioner students to engage with the on-campus students *via* a TPR. Students worked through challenging simulations together, allowing them to practise structured team communication and to develop their professional role identity in a safe learning environment (Rudolph et al., 2017). University of Colorado Boulder, Oral Roberts University and Florida International University are using TPRs to engage their students better no matter where they are in real-time class community and conversation (Kennedy, 2016; Reis et al., 2018; Davey, 2021; Khadri, 2021). In collaboration with Michigan State University, the University of Akureyri in Northern Iceland has used TPRs for several years, allowing students and teachers to meet despite the weather conditions. Users can move around campus, attend lessons, hold meetings, participate in conversations and teach on campus. For example, when people are seated at a table, the TPR also has a seat (Wernbacher et al., 2022).

The use of TPRs in educational settings has focused mainly on increasing accessibility and equity. However, in comparison to other technologies like videoconferencing, the use of TPRs has not yet reached a level of mainstream adoption for educational or professional settings (Lei et al., 2022). The research on the value of TPRs to foster communication and in-class participation is limited. Most studies that have investigated the use of TPRs are conceptual, usually providing proof-of-concepts or small-size case studies in which the robot is tested in a single classroom (Newhart et al., 2016; Weibel et al., 2020). The study by Schouten et al. (2022) was one of the first to test experimentally the effects of robot-mediated human interaction on cooperation and communication in virtual teams, demonstrating that robomorphism is an important concept to consider when studying the effect of human-mediated robot interaction.

### 2.3 The benefits of TPRs in education

Similarly, to other distance learning tools, TPRs can reduce travel costs (Wernbacher et al., 2022). However, several authors have noted that, unlike video conferencing equipment, TPRs offer several advantages over traditional audio- and video conferencing tools (Shin and Han, 2016; Bell, 2017; Keller et al., 2020). Compared to videoconferencing, participants interacting *via* a TPR will use in-presence physical space and experience more social presence, a greater sense of togetherness, and being together in one room (Nowak & Biocca, 2003; Newhart and Olson, 2017). In addition, TPRs enable otherwise impossible interaction with the (remote) environment (Wernbacher et al., 2022). In a work by Schouten et al. (2022), mediated student interaction supported by a TPR was compared with mediated student interaction supported by videoconferencing. The findings indicated that students who used robots experienced stronger feelings of social presence, but they were also perceived as more robotic by their interaction partners (i.e., robomorphism). Yet, the negative effects of the use of a TPR on cooperation through robomorphism is compensated by the positive effects through social presence. The study indicated that robomorphism is an important concept to consider when studying the effect of human-mediated robot interaction. TPRs may thus be applicable as a tool to stimulate classroom participation of absent students, if the use can stimulate the feeling of social presence in a group, and if feelings of robomorphism can be mitigated (Schouten et al., 2022). The main difference between TPRs and traditional cameras is that TPRs can follow actions and sounds to give remote students more natural classroom experience (Khadri, 2021). Videoconferencing students, however, sense being easily forgotten by those physically present, they miss the informal (pre- and after-lesson) classroom conversations and sometimes feel the need to apologize when they want to contribute to what is happening in the physical space (Bell, 2017).

Previous research on TPRs in the educational environment has also demonstrated that TPRs can promote social interaction and collaborative learning through by supporting the social dimension of the learning process. This allows students to more meaningfully participate in (in-person) group work and navigate through remote learning environments (Cha et al., 2017; Soares et al., 2017; Reis et al., 2018; Zhang et al., 2018; Ahumada-Newhart & Olson, 2019; Gonnot et al., 2019; Ahumada-Newhart & Eccles, 2020; Rosenberg-Kima et al., 2020). Bell et al. (2016) examined the feeling of presence of distance students in hybrid teaching using TPRs and video conferencing. Research showed that the mobility of TPR is essential to create a sense of physical presence.

Cheung et al. (2018) highlighted the benefits in implementing Kubi TPRs at the Michigan State University as follows. First, *Persistence and retention*–TPRs help students remain successful in their classes by providing an easy option to overcome challenging circumstances. Second, *Student empowerment*–TPRs empower students who experience setbacks and other serious life challenges, allowing them demonstrate their commitment to learning. Third, *Comparable learning experiences*–TPRs facilitate active participation and peer interactions during class. Fourth, *Easy implementation*–TPRs offer a flexible, affordable, and simple solution that is relatively easy to implement by students and instructors. Fifth, *Minimal impact on instructors*–although instructors require some training to integrate the system into their classroom, its actual use is typically easier than extending deadlines, changing assignments, or scheduling alternate exam days. And sixth, *Empowerment for student-support personnel*–TPRs enable immediate, short-term assistance for students who were experiencing temporary setbacks, empowering personnel to help students who may have had no other options.

To put it shortly, through improved social presence, TPRs improve learner engagement, interest, confidence, motivation in classroom settings, allowing teachers to teach lessons and students to participate in classes from anywhere.

### 2.4 The limitations, threats and challenges related to TPRs in education

The literature highlights the following limitations when employing TPRs in the educational context: 1) *connectivity issues*–bad Wi-Fi or mobile connection would require human assistance; 2) *interaction with the environment*–the inability to physically manipulate objects degrade user experience; 3) *less social presence*–when compared to in-person interaction, TPRs are difficult to use for spontaneous social interaction; 4) *navigation of the robot* (manoeuvrability)—navigating a TPR can be difficult; 5) *narrow field of view* offered by the robot’s camera; 6) *the quality of audio/video* transmission could be improved and can hinder interaction with others; 7) *privacy and legal concerns*; 8) *power consumption and battery*–short battery life can degrade user experience; 9) *inclusion* (10) *cost*–TPRs are relatively costly to purchase and maintain (Bell, 2017; Zhang et al., 2018; Yousif, 2021; Wernbacher et al., 2022). Challenges also exist in combining functionalities such as *adjustable height*, *system stability*, *low-speed control*, and *motion control* for slopes and sudden inclines, and more work is required in these areas to improve the safety of TPRs. It is also expected that the future TPRs will be able to understand spoken orders and have more sophisticated sensors, allowing them to reduce operator workload (Davey, 2021).

One of the most important areas for improvement is to help the operator perceive their own projection. For example, when a student joins a class *via* robot, it can be difficult to manage the audio level so that they are loud enough for people to hear, while not shouting over others in the classroom. This becomes a bigger problem when the class switches between large group and small group interactions, since audio levels must be adjusted repeatedly. Common features in video conferencing tools, like chat and screen sharing, are currently not available or as useful with TPRs, requiring operators to combine multiple tools to achieve the same functionality (Bell, 2017).

Some theoretical and qualitative research results have indicated that educators may have resistance towards using robots due to perceived conflicts with their existing teaching and learning practices (Khanlari, 2014; Ensign, 2017; Karypi, 2018) and low levels of perceived usefulness (Lei et al., 2022). Convincing teachers to accept and adopt novel technologies may require good understanding about the teachers’ perspectives (Milles et al., 2013). Bringing a novel technology with its accompanying methods into existing teaching practices at the universities requires introduction of the technology to the teachers, gathering their feedback, and constructing an implementation plan that considers the needs of teachers (Leoste et al., 2021b, p. 4).

People’s attitudes towards technology are connected to their willingness to adopt this technology (Eagly & Chaiken, 1998; Fazio & Roskos-Ewoldsen, 2005). For example, the prevalence of robotics can cause people to become afraid of becoming marginalised, causing them to form negative attitudes towards robots (Gnambs & Appel, 2019; Dang & Liu, 2021). In addition, negative attitudes, combined with mandatory adoption, can lead to varying usage levels that may discontinue as soon as the compulsion ends (Sinclair & Aho, 2018). The negative attitudes can be reduced and people’s readiness to adopt technologies can be stimulated *via* several approaches, for example, by providing people with adequate information about technologies (Reich-Stiebert et al., 2019) or by highlighting unique human abilities that give humans a competitive edge over machines (Leoste et al., 2021a).

Lei et al. (2022) note that when users approach a new technology such as TPRs, their actions and understanding are shaped by their technological frames. These frames are cognitive models (e.g., assumptions, expectations, and values) that encompass an individual’s perceptions of a technology. Consequently, these frames will shape how an individual could conceptualise the usefulness of a given technology for their purposes, the conditions for its success, and the impact of its use. In turn, when describing the perceptions of the nature and usage of existing robots, Nielsen et al. (2016) collected a wide range of perspectives from participants, such as enthusiasm, cost-savings, concerns about quality of function, and innovation. Research on technological frames shows how a variety of factors can influence the perception of technology by individuals.

In the present study, the authors focused on a small subset of technological frames, specifically individuals’ perceptions about the usefulness, problems and challenges of using TPRs.

## 3 Materials and methods

This study was conducted in the framework of the project *Developing and Implementing Technology-Enhanced Teaching and Learning at Georgian Higher Education Institutions* (DITECH). The aim of the project is to enhance the quality of higher education by developing and implementing technology-enhanced teaching and learning in Georgian *Higher Education Institutions* (HEIs). The main target groups are students, academic personnel and pre-/in-service teachers. All the activities within the project lead to the achievement of the objective of the project–to enhance the quality of higher education by developing and implementing technology-enhanced teaching and learning in Georgian HEIs. The project runs from 15 January 2021 to 14 January 2024. The project is funded under the Erasmus + Capacity Building in the Field of Higher Education (https://www.ditech-erasmus.eu).

**FIGURE 1:**
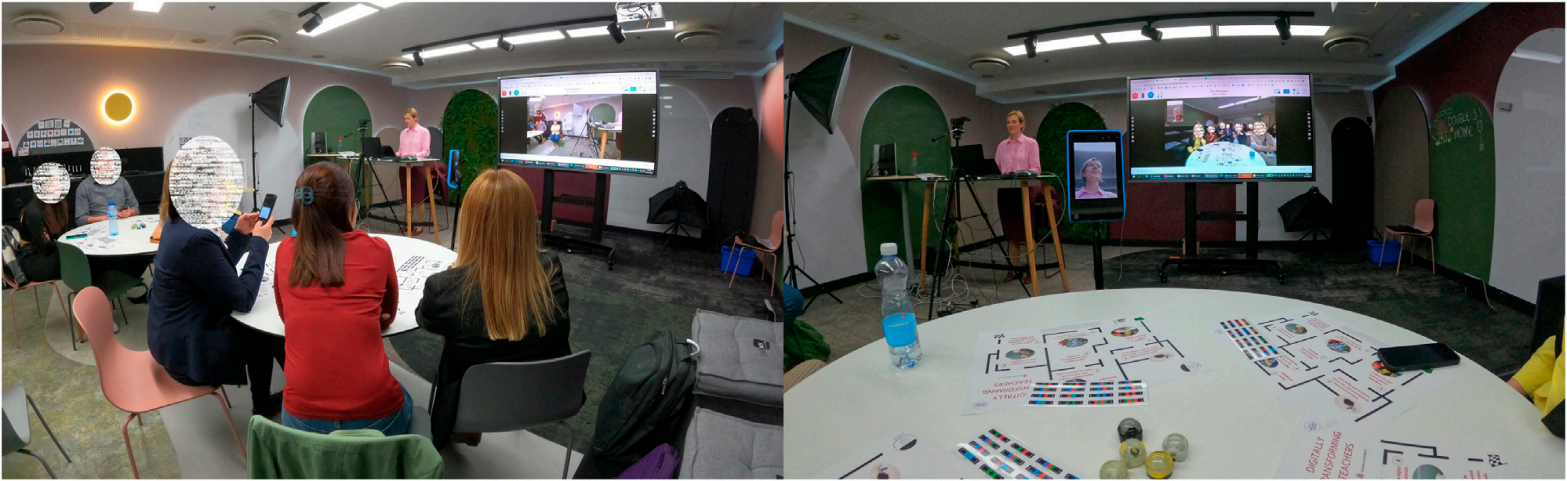
Practical demonstration and experimentation of a TPR.

Within the DITECH project, a professional development training workshop was arranged for the personnel of Georgian HEIs in Tallinn on May 23-27, 2022. The workshop was attended by 22 participants from eight universities in Georgia. The general aim of the workshop was providing participants with advanced views on the ways in which technology can support teaching and learning. TPRs were introduced at this workshop through a 2-h hands-on session, conducted by one of the authors. The workshop was divided into the following parts: conceptualizing the adoption and sustainability stages and factors of technological innovations in higher education (10 min), theoretical introduction, literature overview and use-cases on telepresence robotics (20 min), practical demonstration and experimentation of a TPR (the Double 3 model, as seen on Figure 1). In the practical demonstration, only one TPR was used. Each participant was able to play out the roles of participating in discussion *via* the TPR from a distance (from a different classroom), and that of a physical participant discussing with a telepresent person. As this was the first experience with a TPR for the participants then the discussion scenarios were improvised impromptu, based on the levels of understanding and comfortability of the participants. The main features of the Double 3 telepresence robots are the following: a self-balancing two-wheel chassis with lateral stability control; a 4-h battery, self-driving sensors, two 13-megapixel cameras with pan and tilt capabilities, six microphones, and an amplified speaker. The more exact information is available from the producer’s specs-sheet at https://www.doublerobotics.com/tech-specs.html.

### 3.1 Data collection and analysis

Data for this study was collected using an online Google Forms questionnaire (Appendix 1). A link to the survey was distributed to an initial pool of 22 participants of the TPRs workshop after the workshop at the beginning of June 2022. Two additional reminders about the survey were sent to the workshop participants in the middle of June 2022. In total 16 (sixteen) participants filled in the survey.

The questionnaire consisted of 21 questions divided into four main sections: 1) a background information section that recorded demographic and professional characteristics; 2) experience and perception section which recorded the participants previous experience with TPRs, their initial reaction about the TPRs, the usefulness of TPRs as a faculty extender, the opportunities, threats, weaknesses and challenges of using TPRs as well as the value of experience and comfort when they personally used it during the workshop; 3) questions about the future role of TPRs; and 4) questions about the participants’ training needs related to TPRs.

The questionnaire included closed-ended questions (dichotomous or two-point questions), scaled questions (such as the Likert scale) and open-ended questions, accompanying a Likert question or having individual content.

Data was transferred from Google Forms to MS Excel for data organization and analysis. MS Excel was also used for working with comments and results of open-ended questions. A descriptive analysis has been applied to the quantitative data. The responses to the open-ended question were analysed by two researchers, using content analysis. The descriptive statistics were analysed and discussed in light of the literature review.

### 3.2 Sample

A final sample group (N = 16) consisted of 11 women and 5 men. Participants ranged in age from 21 to 60+: 3 respondents in the range of 21–30, 5 in the range of 31–40, 5 in the range of 41–50, 2 ranged from 51 to 60, and one was more than 61. Most of the study participants (10) were in the age range of 31–50 years.

Based on positions, the participants were professors (2), associate professors (3), lecturers 7) and administrative personnel (4). All but one of the participants (administrative personnel) had experience as a university teacher, ranging from one to more than 20 years. Three people had educational experience more than 20 years, one person had experience between 16 and 20 years, 3 persons between 11 and 15 years, 4 persons between 6 and 10 years, 3 persons between 3 and 5 years and one person between 1 and 2 years. One person did not have educational experience. The sample demographics is also presented in Table 1.

**TABLE 1:** The sample demographics (gender, age, position, educational experience).

Gender	N	Age	N	Position	N	Educational experience	N
F	11	21–30	3	Professor	2	1–2 years	1
M	5	31–40	5	Ass. Professor	3	3–5 years	3
		41–50	5	Lecturer	7	6-10-year	4
		51–60	2	Adm. Personnal	4	11-15-year	3
		60+	1			16–20 years	1
						More than 20 years	3
						No experiencce	1

The participants of the survey were of different disciplines: eight were from educational sciences including educational administration and management, and technology-enhanced learning. Two respondents were from philology, and there was one respondent from each of the following disciplines: economics, business administration, mathematics, computer sciences and information technologies. One respondent did not answer this question. Thus, educational sciences was the most represented discipline area in this study. A majority of the sample was novice and inexperienced; 14 respondents rated their skills related to TPRs (TPR) as novice and two respondents as intermediate. Only one person had previous limited experience with TPRs (see Table 2).

**Table 2:** The sample demographics (disciplinary background, experience with TPR).

Disciplinary background	N	Experience with TPR	N
Educational sciences	5	Novice	14
Educational administration	1	Intermediate	2
Educational management	1		
Technology-enhanced learning	1		
Philology	2		
Economics	1		
Business administration	1		
Mathematics	1		
Computer sciences	1		
Information technologies	1		
No answer	1		

## 4 Results

Our goal was to clarify the perspectives of implementing TPRs in higher education institutions. For these purposes, we made a literature review (Section 2) and examined university personnel’s perceptions about TPRs by using an experimental workshop. Next, we will present the results of our analysis of the university personnel’s perceptions (section 4.1). In addition, based on the synthesis of literature review and university personnel’s perceptions, we will describe the benefits, opportunities, weaknesses, threats and challenges of TPR proliferation in higher education.

### 4.1 University personnel’s perceptions about TPRs

**FIGURE 2:**
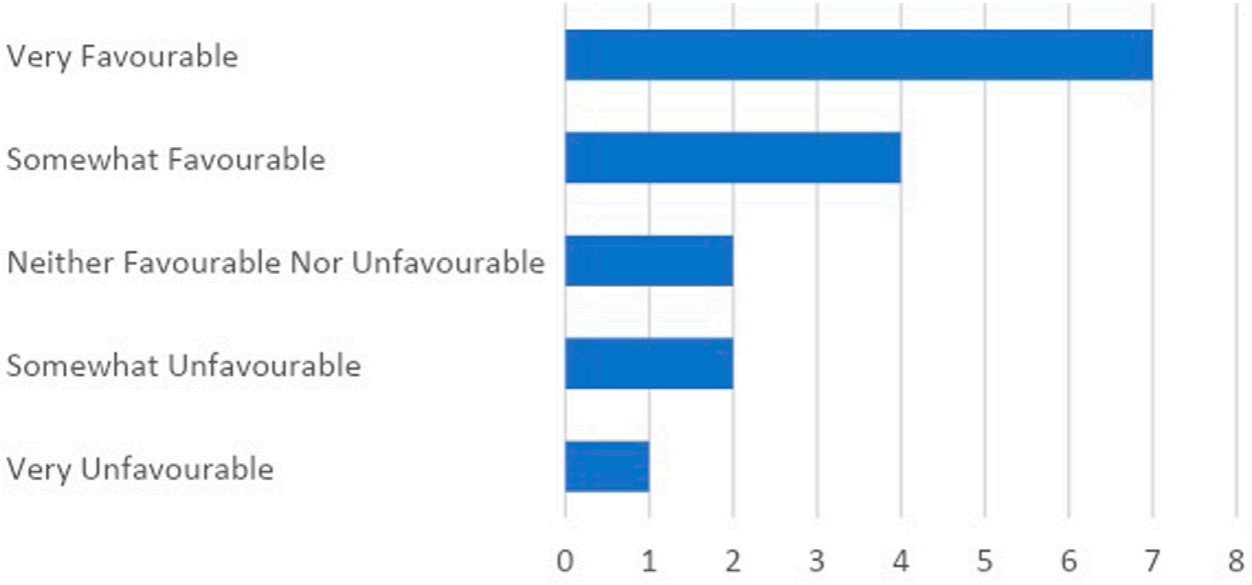
Teachers’ initial assessment of TPRs.

The majority of participants 11) marked that their initial perception of TPRs was favourable, while four participants viewed them unfavourably and two were neutral (Figure 2). Participants’ additional comments expressed their interest in the use of TPRs in training; for example, the comments included: “*My experience was the first time and I have a desire to experience again*”, “*Interesting*”, “*That was something new and fresh for me*”, “*It was very actual and innovative*”, “*The one moving in the classroom can be used, while the table playing ones need more time to design activities with*” and “*I strongly believe that this is a future of education*”.

All of the participants found TPRs either useful or very useful when used as faculty extenders. Most of them considered their experience of using a TPR as very special, interesting, exciting, unusual, fun, and also very important and useful. Some participants noted that they had acquired new information about TPRs, information that they did not have before. In their opinion, TPRs would be good tools in case of necessity. However, they also mentioned that the workshop offered too little time for interaction. Some answers that illustrate the responses:

This is such big news for the Georgian reality that it was very exciting for me. I would like to be one of the first users in our country who will start using these types of robots in educational and scientific fields.

I personally got information about the telepresence robots that I did not have before.

It was very interesting and fun, also very important and useful.

Most participants noted that they could not use the newly acquired information about TPRs in their daily work and activities as of yet, because the equipment is not available at their organizations. However, they believed that they could use them in the future. For example:

I do not think that my daily activities in Georgia will allow me to make massive use of the information received about this type of robot, but I will definitely share this knowledge with my colleagues, students and all interested parties.

As telepresence robots are very expensive, it will be difficult to use them in my daily life.

Not yet, but definitely I will use it in the future.

The level of comfort of using TPRs was found by participants to be interesting, unusual, comfortable, motivating, pleasant and challenging. However, some of them noted that an adaptation period is needed. For example, “*I am good at technology. So I think it would be challenging for me but quite pleasuring”* and *“I will feel very motivated to use the TPR as I am open to any new technological novelty”*. In addition:

I think I will need an adaptation period too, although this period in my case involves several test uses. Beyond that, I think it will be very comfortable and interesting to use it during various activities in the academic and non-academic space. Which in turn allows me to make some sort of observation and research in relation to the study of public acceptance.

All workshop participants except one noted that they became more interested in learning more about TPRs after this workshop. The participants found that the training was very useful, the use of intelligent robots in education will be the future and therefore we would need to learn much more about them. They hoped to have more opportunities to learn how to use TPRs.

We also examined which topics about TPRs were considered necessary by the participants (Table 3). The topic the participants valued the most was “*Use of intelligent robots in education*” (Average rating 3.63), followed by “*Ethical aspects of using robots”* (3.5), *“Introduction of the world of robotics”* (3.31) and *“Work safety with collaborative robots”* (3.31)*, “The role of robots in shaping a sustainable world*” (3.25), and “*Technical aspects of intelligent robots*” (3.19).

**TABLE 3:** Learners’ evaluation of the required course topics.

Topic	Average rating
Use of intelligent robots in education	3.6
Ethical aspects of using robots	3.5
Introduction of the world of robotics	3.3
Work safety with collaborative robots 3.3	3.3
The role of robots in shaping a sustainable	3.25
Technical aspects of intelligent robots	3.19

### 4.2 What are the benefits, opportunities, weaknesses, threats and challenges of proliferation of TPRs, from the point of view of university personnel?

There were no significant differences in participants’ perceptions on TPRs, based on their gender, age, background, work experience and position. Of course, the sample was quite small to make such generalisations. There was some overlapping of subcategories in different categories. For example, a number of benefits were also identified as opportunities and a number of weaknesses as threats or challenges.

The *benefits* associated with TPRs have, according to the participants, mainly related to the replacement of physical presence and the creation of a sense of social presence. It was mentioned that it allows a teacher or student to be in several places at the same time and carry out studies or teaching while travelling. Some participants also mentioned that it could make learning more attractive to students, give a chance to students to improve different skills (creativity, analytical skills, decision-making, etc.), allow working without attendance or just be useful in modern times and face the problems and challenges we face in the 21st century. Four people did not give an answer to this question (see Table 4). The following citations from the workshop participants illustrate their opinions about the benefits of TPRs:

**TABLE 4:** Benefits of TPRs.

Main categories	Subcategories	Occurrences (N)
Benefits	Replacement of physical presence	3
	Creation of a sense of social presence	3
	To be in several places at the same time	2
	Opportunity to teach while travelling	2
	Making learning more attractive	1
	To improve student skills	1
	Allows working without attendance	1
	To face problems/challenges of the 21st century	1
	Useful in modern times	1
	No answers	4

I think robots can be a very practical and innovative finding to face the problems and challenges we face in the 21st century. In order to increase technology-enhanced teaching in higher education institutions, in order to be able to be in several places at the same time, in order for our attendance to be perceived in a physical way.

It is beneficial, when there are two parallel meetings planned in different countries and the presence of the professor is required in both places.

The main *opportunities* of TPRs are related to providing a social presence, the possibility to be in different places at the same time, improving the quality of teaching, allowing participation in classes from remote areas and from international settings, supporting students of special needs, enriching classroom experience and increasing the level of interest among audience (see Table 5). For example:

**TABLE 5:** Opportunities of TPRs.

Main categories	Subcategories	Occurrences (N)
Opportunities	Provide social presence	4
	To be in several places at the same time	2
	Improve quality of teaching	2
	Allowing participation from remote areas	1
	Allowing international participation	1
	Supporting students of special needs	1
	Enriching classroom experience	1
	Providing classes in the peripheral areas	1
	Increasing the level of interest	1
	No answers	2

These types of robots give the same physical feeling of being at specific meetings, seminars, training, conferences and so on, as well as the ability to be in different places at the same time, use technology in educational or scientific activities and more.

Several participants in the workshop were unable to point out *weaknesses* of the TPRs because they did not have sufficient knowledge and skills to evaluate them. The main aspects highlighted were that they are too expensive, lack of knowledge, skills, experience and expertise in higher education institutions, lack of necessary infrastructure, connectivity issues, lack of funding, risk of failure, slowness and lack of human touch. It was also suspected that, as the TPR is a machine, any surprises could occur (see Table 6).

**TABLE 6:** Weaknesses of TPRs.

Main categories	Subcategories	Occurrences (N)
Weaknesses	High cost	3
	Lack of knowledge and skills	3
	Lack of necessary infrastructure	3
	Lack of experience	2
	Lack of expertise in HEIs	1
	Risk of failure	1
	Slowness	1
	Connectivity issues	1
	Lack of funding	1
	Lack of human touch	1
	Not comparable with physical presence	1
	No answers	4

One participant noted:

First and foremost, it is probably the lack of field specialists in each higher education institution. It is also important to have this type of experience across the country. Adapted environment for robots, high-speed internet and most importantly - funding for this type of technical equipment.

Another mentioned, “*You are still a machine and maybe you can’t be charged or someone will turn you off”.*

Seven participants were unable to identify *threats*. Other participants most often mentioned failures of robots, high cost, lack of necessary infrastructure and funding, and availability. It was also mentioned that the adaptation period with robots would be long in Georgia (see Table 7). For example, the participant noted:

**TABLE 7:** Threats of TPRs.

Main categories	Subcategories	Occurrences (N)
Threats	Failures of robots	3
	High cost	2
	Lack of funding	2
	Lack of infrastructure	1
	Availability	1
	Long adaption period	1
	No answers	7

Apart from financial support for the example of Georgia, as far as I can see, there is a great danger that the society will not be able to involve these types of robots in various fields of activity. Anyway, I think a pretty big, long adaptation period will be going through.

The biggest *challenges* in using TPRs in education are their high cost, as they are too expensive for Georgian universities to acquire. Cost issues are followed by failures of robots, acceptance of TPRs, lack of knowledge and skills, limited experience with TPRs, lack of funding and necessary infrastructure, needs for technical support, services and personnel training, and fear of technology (see Table 8). For example, the participants noted:

**TABLE 8:** Challenges of telepresence robots.

Main categories	Subcategories	Occurrences (N)
Challenges	High cost	5
	Failures of robots	2
	Acceptance of telepresence robots	2
	Limited experience	2
	Lack of funding	1
	Lack of infrastructure	1
	Needs for technical support and services	1
	Training needs	1
	Lack of knowledge and skills	1
	Fear of technology	1
	No answers	2

I think our Georgian society lags far behind Estonia in terms of technological development. Producing these types of robots requires a lot of money, in addition to funding; it is also noteworthy that our (university space) personnel will need serious technical training, skills and knowledge.

One thing is to buy such a robot and start using it, but the second and no less important is to be able to technically support it and provide the necessary services in case of damage/technical malfunction to technically operate it.

## 5 Conclusion and discussion

Our study examined the perspective of implementing TPRs in higher education institutions, obtaining data from the existing literature and written feedback, provided by the personnel of Georgian universities. Our first research question was to establish the current state of existing literature on using TPRs in higher education, and for this purpose, we conducted a literature review. The review indicated that, in principle, the research field of TPRs is 16 years old, with major contributing countries being the United States, Japan and Canada, and other technologically advanced countries. In education, the early works have been studying TPRs in specific circumstances (e.g., with children with autism spectrum disorder) and the papers that focus on TPRs in general education have seen the growth in their numbers during the recent years, coinciding with the COVID-19 years when there was higher demand for distance learning solutions. However, while the number of works regarding TPRs in education is respectable, there is a shortage of longitudinal and comparative works with large samples, studying, *inter alia*, the influence of TPRs on student learning outcome or teacher workload.

According to literature, TPRs are seen as beneficial in maintaining the continuity of in-person education in situations where a student’s physical presence is obstructed. In addition, especially when compared to computer-based distance learning methods, TPRs are seen as useful for keeping students’ (or teachers’) social presence, allowing them to employ a wider range of classroom discussion nuances (including unofficial discussion between the actual lesson). A number of limitations and challenges are listed in the literature, most of them related to the technical limitations of the current generation of TPRs or relevant infrastructure (e.g., limitations in video, audio, gaps in presence due to insufficient internet speed, limited autonomous behaviour of the robotic body, etc.), less so considering the ethical and legal aspects of possible widespread use of TPRs in education. In addition, the literature refers to the need to overcome teachers’ sceptical attitudes towards this novel technology that could hinder implementation efforts.

Our second and third research questions required us to examine the university personnel’s perceptions about TPRs, using the example of personnel from eight Georgian universities, after a short 2-h hands-on exposure to this technology. Based on their perceptions, we strived to describe the benefits, opportunities, weaknesses, threats and challenges of TPR proliferation in higher education. The results of our study suggest that the working principles of TPRs and their usefulness are easy to understand after a 2-h hands-on workshop session–a format that seems to be enough for familiarising university personnel with TPRs, encouraging them to look for more information and making them believe that their future teaching practices will include TPRs. In general, TPRs are considered as an empowering tool, allowing teachers and students to be present at various physical locations, making it easier to maintain the continuity of education while travelling. Compared to computer-based distance learning methods, TPRs are seen as important in keeping social relations between classroom participants (both students and teachers) and in enhancing students’ 21st century skills.

Our data indicates that the initial bias towards TPRs is positive among university personnel, making it thus easier to introduce the technology when financially possible and feasible. However, according to gathered data, the participants perceived TPRs as a novel technology, characterised as being exiting, unusual and fun–although important and useful, suggesting that any attempt to implement TPRs should include a teacher training course that aims at developing skills and knowledge, both pedagogical and technological, necessary for successful use of TPRs in the classroom settings. Indeed, the lack of previous exposure to TPRs, indicated by the participants’ little understanding about its weaknesses, combined with the threats and challenges they listed, leads us to believe that successful and sustainable implementation of TPRs requires a proper and meticulous planning process. This planning process should allocate financial resources for devices and infrastructure; provide a training course for developing necessary skills, knowledge and expertise in the implementing institution, while taking into consideration the specific problem areas of TPRs (e.g., slowness or risk of failure). This requirement for thorough planning of the implementation process of an educational innovation is similarly highlighted by Leoste et al. (2021c), and, as is the case with implementation of innovative solutions in education in general, should go hand in hand with policy planning, often requiring large investments into the sector. We conclude that, in principle, the university personnel is ready to accept TPRs as a teaching and learning tool that, although novel, is useful (or even indispensable) in certain scenarios. However, despite being a promising technology, the information about its influence on student learning outcome and teacher workload is still scarce and more studies are needed to overcome this gap in knowledge.

This study has its own limitations that can have an impact on its results. First, our intervention used a small sample of personnel (16 persons) from eight Georgian universities, making it difficult to understand which disciplines would benefit the most from TPRs. For better quality data, a larger sample is needed with clearly differentiated discipline boundaries. In addition, involving lecturers and students of different disciplines would provide additional information. Another problem of our study was the fact that it was carried out as a one-time workshop session. While providing valuable information about university personnel’s attitudes, this information has limited usefulness for understanding the real-classroom usage aspects. We are currently planning a larger longitudinal study (with planned duration from autumn 2022 to spring 2023) that should address these issues. In this study, ten higher education teachers will use TPRs in their lessons (with more than 300 students) and we will strive to create understanding about the circumstances that justify the use of TPRs in higher education, and about features that are required from TPRs for these purposes.

## Data Availability

The original contributions presented in the study are included in the article/supplementary materials, further inquiries can be directed to the corresponding author.

## Appendix Use of telepresence robots in the teaching process

### Demographics:

• Gender (Female/Male)
• Age (21-30, 31-40, 41-50, 51-60, 61+)
• Position (professor, associate professor, lecturer, researcher, administrative staff, other)
• Experience as a university teacher (1–2 years, 3–5 years 6–19 years 11–15 years, 16–20 years, more than 20 years, not applicable)
• Educational/disciplinary background

#### Experience and perceptions

• Do you have previous experience with telepresence robots? (None, Limited, Competent, Experienced). Comments
• What is/was your initial reaction about the telepresence robots? (Very Favourable 5 4 3 2 1 Very Unfavourable). Comments.
• Do you think a robot could be useful as a faculty extender? (Very useful 5 4 3 2 1 Not useful). Comments.
• Please provide an example of what you found to be the most beneficial aspect of using the telepresence robots.
• Please provide an example of what you found to be the biggest challenge of using the telepresence robots.
• Please provide examples of what you found to be the weaknesses of using the telepresence robots.
• Please provide examples of what you found to be the opportunities of using the telepresence robots.
• Please provide examples of what you found to be the biggest threats of using the telepresence robots.
• What was the value of this experience for you personally when you used it?
• How would you describe your comfort level using the telepresence robot or with one of your peers using the telepresence robot?

#### Training needs related to telepresence robots

• Please evaluate, which training course topics could be the most needed and interesting for you? (Not Useful 1, 2, 3, 4 Very Useful). Comments.
  o Introduction of the world of robotics  
  o The role of robots in shaping a sustainable world
  o Use of intelligent robots in education 
  o Technical aspects of intelligent robots  
  o Ethics aspects of using robots  
  o Work safety with collaborative robots
  o Something else (Please specify).
• Did you become more interested in learning more about telepresence robots after this workshop? (Yes/No)
• How much can you use what you learned about telepresence robot in your daily work and activities?
• Is there anything else that you’d like us to know?
